# Breast carcinoma-amplified sequence 2 regulates adult neurogenesis via β-catenin

**DOI:** 10.1186/s13287-022-02837-9

**Published:** 2022-04-11

**Authors:** Hsin-Hsiung Chen, Hao-Yu Lu, Chao-Hsin Chang, Shih-Hao Lin, Chu-Wei Huang, Po-Han Wei, Yi-Wen Chen, Yi-Rou Lin, Hsien-Sung Huang, Pei-Yu Wang, Yeou-Ping Tsao, Show-Li Chen

**Affiliations:** 1 Graduate Institute of Microbiology, College of Medicine, National Taiwan University, 7F, No1, Sec. 1, Jen-Ai Rd., Taipei 100, Taiwan; 2 Graduate Institute of Brain and Mind Sciences, College of Medicine, National Taiwan University, No. 1, Section 1, Jen Ai Road, Taipei 100, Taiwan; 3 Department of Ophthalmology, Mackay Memorial Hospital, No. 92, Sec. 2, Chung Shan North Road, Taipei 104, Taiwan

**Keywords:** BCAS2, β-catenin, Adult neurogenesis, Sox2^+^, DCX^+^ immature neurons, AAV-DJ8

## Abstract

**Background:**

Breast carcinoma-amplified sequence 2 (BCAS2) regulates β-catenin gene splicing. The conditional knockout of BCAS2 expression in the forebrain (BCAS2 cKO) of mice confers impaired learning and memory along with decreased β-catenin expression. Because β-catenin reportedly regulates adult neurogenesis, we wondered whether BCAS2 could regulate adult neurogenesis via β-catenin.

**Methods:**

BCAS2-regulating neurogenesis was investigated by characterizing BCAS2 cKO mice. Also, lentivirus-shBCAS2 was intracranially injected into the hippocampus of wild-type mice to knock down BCAS2 expression. We evaluated the rescue effects of BCAS2 cKO by intracranial injection of adeno-associated virus encoding BCAS2 (AAV-DJ8-BCAS2) and AAV-β-catenin gene therapy.

**Results:**

To show that BCAS2-regulating adult neurogenesis via β-catenin, first, BCAS2 cKO mice showed low SRY-box 2-positive (Sox2^+^) neural stem cell proliferation and doublecortin-positive (DCX^+^) immature neurons. Second, stereotaxic intracranial injection of lentivirus-shBCAS2 knocked down BCAS2 in the hippocampus of wild-type mice, and we confirmed the BCAS2 regulation of adult neurogenesis via β-catenin. Third, AAV-DJ8-BCAS2 gene therapy in BCAS2 cKO mice reversed the low proliferation of Sox2^+^ neural stem cells and the decreased number of DCX^+^ immature neurons with increased β-catenin expression. Moreover, AAV-β-catenin gene therapy restored neuron stem cell proliferation and immature neuron differentiation, which further supports BCAS2-regulating adult neurogenesis via β-catenin. In addition, cells targeted by AAV-DJ8 injection into the hippocampus included Sox2 and DCX immature neurons, interneurons, and astrocytes. BCAS2 may regulate adult neurogenesis by targeting Sox2^+^ and DCX^+^ immature neurons for autocrine effects and interneurons or astrocytes for paracrine effects.

**Conclusions:**

BCAS2 can regulate adult neurogenesis in mice via β-catenin.

**Supplementary Information:**

The online version contains supplementary material available at 10.1186/s13287-022-02837-9.

## Background

Breast carcinoma-amplified sequence 2 (BCAS2), a 26-kD nuclear protein, is a negative regulator of the tumor-suppressor p53 [1, 2]. BCAS2 is essential for *Drosophila* viability [3] and regulates *Delta*–*Notch* signaling via *Delta* pre*-*mRNA splicing *in Drosophila* wing development [4]. Additionally, BCAS2 can regulate β-catenin expression for dendrite growth of mouse neurons [5]. BCAS2 is related to fertility: in males, it regulates alternative splicing of *Dazl*, an essential gene for germ cell survival [6], and in females, maternal BCAS2 can maintain genome integrity in early embryonic development [7]. Hence, BCAS2 functions in development.

BCAS2 is involved in the Prp19–CDC5L spliceosome complex that regulates RNA splicing [8]. We found that BCAS2 upregulates β-catenin splicing [9], and BCAS2 conditional knockout (cKO) in the postnatal forebrain of animals caused microcephaly-like features, dendritic malformation, and poor learning and memory phenotypes due in part to downregulated β-catenin [5]. Disrupted Wnt/β-catenin signaling is involved in several neurodegenerative diseases such as Alzheimer disease and autism [10, 11]. Microarray assay revealed decreased mRNA expression of BCAS2 in the hippocampus of people with Alzheimer disease [12]. In addition, genetic variants of the BCAS2 gene linkage region (1p13.2) were associated with the risk of autism [13], which implies that BCAS2 is associated with neural-related functions.

The association between Wnt⁄β-catenin signaling and adult neurogenesis is well documented: β-catenin reportedly regulates hippocampal neurogenesis, proliferation of neural progenitors, dendrite formation, and neuron maturation [14–17]. Adult neurogenesis is restricted to two distinct brain regions in the central nervous system: the subgranular zone (SGZ) in the dentate gyrus (DG) of the hippocampus and the subventricular zone of the lateral ventricle [18, 19]. It involves proliferating expansion and cell fate specification of adult neural progenitors as well as their subsequent differentiation and maturation in the existing neuronal circuitry [18]. It also participates in cognitive functions (learning and memory) [20].

Previously, we found that BCAS2 regulates β-catenin [5, 9]. SRY-box 2 (Sox2) and doublecortin (DCX) are widely used as markers for neurogenesis [21]. To determine whether BCAS2 plays a role in adult neurogenesis via β-catenin, we characterized BCAS2-regulating Sox2^+^ neural stem cell (NSC) proliferation and increasing the number of DCX^+^ immature neurons by using three different methods: (1) characterization of BCAS2 cKO in mice, (2) stereotaxic intracranial injection of lentivirus-shBCAS2 into the hippocampus to knock down BCAS2 expression, and (3) rescue by adeno-associated virus (AAV-DJ8)-BCAS2 gene therapy via intracranial injection in BCAS2 cKO mice. Moreover, we used AAV-β-catenin gene therapy in BCAS2 cKO mice to rescue adult neurogenesis. BCAS2 regulated Sox2^+^ NSC proliferation and increased the number of DCX^+^ immature neurons in the hippocampus via β-catenin.

## Materials and methods

### BCAS2 cKO mice

BCAS2 cKO mice were generated by the Cre/loxP system as described [5]. Control mice were CaMKIIα-iCre^+^ mice. Genotyping was analyzed by PCR of genomic DNA extracted from mouse tails.

### Cell culture and transfection

HEK293T (human kidney cell line) cells were grown in DMEM containing 10% fetal bovine serum and transfection by using jetPRIME (Polyplus transfection, NY, USA) following the manufacturer’s guidelines.

### Adeno-associated virus (AAV) production and purification

For pAAV-BCAS2-GFP plasmid construction, the inserted BCAS2-GFP was purified by PCR and ligated with the pAAV-MCS vector. Both the GFP vector control and BCAS2-carrying plasmids were co-transfected with pHelper and pAAV-DJ8 into HEK293T cells for 72 h. Cell pellets were frozen and thawed between liquid nitrogen and a 37 °C water bath for 10 cycles to obtain supernatant containing AAV-GFP or AAV-BCAS2 virus. For AAV purification, AAV-containing supernatant was adjusted to 1.37 g/mL by adding CsCl powder followed by ultracentrifugation. Virus suspension was aliquoted to avoid repeated freezing and thawing for indefinite storage. For pAAV-MCS-β-catenin-3xFlag plasmid construction, β-catenin (CTNNB1) gene was fused with Flag tag, then amplified by PCR from p3XFlag-CMV14-β-catenin and ligated with the pAAV-CMV-MCS vector by restriction enzyme sites (CalI and SalI), generating the plasmid pAAV-MCS-β-catenin-3xFlag; the recombinant virus was named AAV-β-catenin.

### Stereotaxic injection in mouse brain

Mice were anesthetized with 2.5% avertin (2,2,2-tribromoethanol, Sigma-Aldrich, St. Louis, MO, USA) and placed in the stereotaxic apparatus. The animal’s head needed to look horizontal and be symmetrical to the ear bars. Head skin was incised with small surgical scissors and separated by surgical hooks to keep the surgical area open. The positions of the x and y coordinates of the bregma were measured, and the coordinates of the target injection area were calculated. For DG injection, coordinates relative to the bregma were for anterior and posterior: − 2.1 mm; lateral: ± 1.9 mm; ventral: − 2.5 mm. The injection syringe with an adequate volume of virus (AAV or lentivirus) was placed into the holder of the stereotaxic arm, and the syringe was slowly lowered to the indicated injection site for injecting AAV. The scalp was stitched closed immediately after virus injection, and animals were kept warm until they fully recovered.

### BrdU administration

To evaluate cell proliferation, mice were injected with bromodeoxyuridine (BrdU, Sigma-Aldrich, St. Louis, MO, USA) intraperitoneally (50 mg/kg in sterile phosphate-buffered saline). At 24 h after the last BrdU injection, mice were killed. Fixed brains were prepared as serial vibratome sections for immunofluorescence analysis (IFA).

### Western blot analysis

Protein lysates were extracted from cells or brain tissues and underwent western blot analysis with the antibodies β-catenin (BD, San Jose, CA, USA; 1:5000), p-GSK3β (BD; 1:5000), β-actin (Sigma-Aldrich, St. Louis, MO, USA; 1:10000), BCAS2 (Proteintech, Rosemont, IL, USA; 1:10000), Flag (Sigma-Aldrich; 1:10000), anti-mouse IgG (Sigma-Aldrich; 1:10000), anti-rabbit IgG (Sigma-Aldrich; 1:10000).

### Brain slice preparation

Mice were anesthetized with avertin (2,2,2-tribromoethanol, Sigma-Aldrich) and then transcardially perfused with phosphate-buffered saline and 4% paraformaldehyde (PFA). Brains were isolated and post-fixed with 4% PFA for 2 days at 4 °C. PFA-fixed brains were sectioned coronally at 40 μm by using a vibratome and stored at 4 °C.

### Immunofluorescence assay (IFA)

The 40-μm floating sections (series of every 12th section from the hippocampus) underwent IFA as described (4) and were incubated with the antibodies β-catenin (BD; 1:500), BCAS2 (Proteintech, Rosemont, IL, USA; 1:10000), Cre (Abcam, Cambridge, UK; 1:500), Sox2 (Abcam; 1:500), BrdU (Abcam; 1:500), NeuN (Millipore, NJ, USA; 1:1000), DCX (Millipore, Darmstadt, Germany; 1:500), GFAP (Sigma; 1:500), GAD67 (Abcam; 1:250), and cyanine Cy3 goat anti-mouse IgG, Alexa Fluor 488 donkey anti-rabbit IgG, Alexa Fluor 647 goat anti-rat igG, Alexa Fluor 488 donkey anti-rat (all Jackson ImmunoResearch PA, USA; 1:1000) (Additional file 2: Table S1). Images were captured by using a Leica TSC SP5 confocal microscope.

### Stereological analysis

For quantification of immunostained-positive cell number, total of four pieces from every 12th section of 40-μm vibratome sections containing hippocampus were chosen for IFA. The images were visualized under a Leica TSC SP5 confocal microscope (Leica Microsystems, Wetzlar, Germany) with a 40X oil-immersion objective lens. Positive-stained cells on every section were counted visually; the number of positive-stained cells throughout the hippocampus was calculated, and the cell number of every section was multiplied by the number of sections (one-in-12 series of coronal sections). Cell number per DG = (sum of counted cells in every section) × 12 [21].

### Statistical analysis

Statistical comparisons involved two-tailed Student’s *t* test and use of GraphPad Prism 6. Data are represented as mean ± SEM. *P* < 0.05 was considered statistically significant.

### Study approval

All procedures of the animal experiments were reviewed and approved by the Institutional Animal Care and Use Committee at the College of Medicine, National Taiwan University (IACUC), and all experiments were performed in accordance with the approved relevant guidelines and regulations. All experimental mice were housed in the animal center under a 12-h light/dark cycle with free access to food and water.

## Results

### Reduced adult neurogenesis in hippocampus of BCAS2 cKO mice

**Fig. 1:**
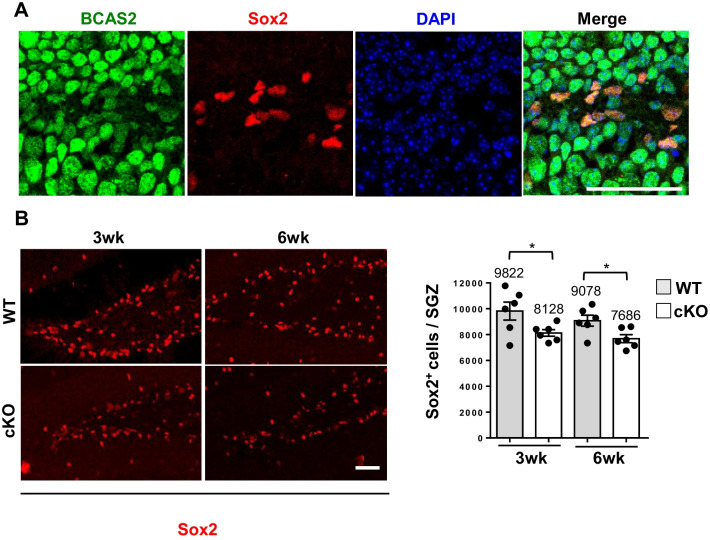
BCAS2 co-localizes with Sox2 in the hippocampus of wild-type (WT) mice. The 10-μm forebrain paraffin sections of 3-week-old WT mice were used for immunofluorescence analysis (IFA) by confocal microscopy. Scale bar: 50 μm. **A** BCAS2 (green) co-expressed with Sox2^+^ neuron stem cells (NSCs) (red) in the neurons of the subgranular zone (SGZ). **B** Representative images of Sox2^+^ in the SGZ of mice at age 3 and 6 weeks. Sox2^+^ NSCs in mice. Scale bar: 50 μm. Right: quantification of Sox2^+^ cells in SGZ. Number inside box: number of mice for test per group. Data are mean ± SEM by Student’s *t* test. *P < 0.05; **P < 0.01

Previously, we found that BCAS2 was a nuclear protein and regulated β-catenin splicing [2, 5, 9]. The Wnt/β-catenin signaling pathway participates in hippocampal neurogenesis [14, 15, 22]. Sox2 is also a nuclear protein widely used to identify NSCs in the SGZ and DCX as a marker for stem cells differentiating into immature neurons [23, 24]. To address whether BCAS2 plays a role in adult neurogenesis, we investigated the effect of BCAS2 on Sox2^+^ NSC proliferation in the SGZ of the DG and DCX^+^ immature neurons in the hippocampal DG region of mice. First, we examined the co-localization of BCAS2 and Sox2 in coronal brain sections from wild-type mice (WT) at age 3 weeks showed BCAS2 co-localized with Sox2 in the nuclei of NSCs in the SGZ (Fig. 1A). The volume of DG is smaller in the forebrain of BCAS2 cKO than WT mice at the age of 12 weeks [5]. We then examined the number of Sox2^+^ NSCs in the SGZ of BCAS2 cKO mice driven by CaMKIIα-iCre to deplete BCAS2. The DG volume was smaller in cKO mice than their age-matched WT counterparts at age 3 and 6 weeks (Additional file 1: Figure S1A; WT vs. cKO: 0.42 vs. 0.36 mm^3^ at 3 weeks, *P* < 0.05; 0.46 vs. 0.38 mm^3^ at 6 weeks, *P* < 0.01), which was consistent with our previous findings at age 12 weeks [5]. Additionally, at age 3 and 6 weeks, the Sox2^+^ NSC number in the SGZ was significantly lower in BCAS2 cKO than WT mice (Fig. 1B; WT vs. cKO: 9822 vs. 8128 cells at 3 weeks, *P* < 0.05; 9078 vs. 7686 cells at 6 weeks, *P* < 0.05). Our data also showed that Sox2 expression was lower at age 6 weeks of WT mice than 3 weeks; those were consistent with the previous report show the decreased Sox2 expression in aged mice [25]. Thus, BCAS2 plays a role in maintaining Sox2^+^ NSCs in the SGZ. The process of neurogenesis starts from Sox2^+^ NSC proliferation, and then, symmetrically dividing into one for self-renewal NSCs for maintenance of stem cell pool, the other for activating NSC to expand into neural progenitor cell (NPC) following differentiation [26, 27]. Loss of BCAS2 induces apoptosis in p53 wild-type cancer cells and causes cell arrest in p53-null cancer cells [1]. Now we demonstrated BCAS2-inducing adult neurogenesis. In order to verify whether the decrease of Sox2^+^ NSC in BCAS2 cKO hippocampus results from the apoptosis of NSC. The terminal deoxynucleotidyl transferase (TdT)-mediated dUTP nick-end labeling (TUNEL) and activated caspase-3 by IFA [28] were performed for apoptosis analysis. Firstly, the positive control for the TUNEL assay, DNase-treated sections (5 μg/ml DNaseI), showed all nuclei positive, but not in negative control (without DNase treatment) (Additional file 1: Figure S1D). The expression of TUNEL was low both in WT and in BCAS2 cKO mice and showed no significant difference between them (Additional file 1: Figure S1E). Similarly, the expression of activated caspase-3 also showed no significant difference between WT and BCAS2 cKO mice (Additional file 1: Figure S1F). In sum, the reduced Sox2^ +^ NSC number of BCAS2 cKO mice (Fig. 1B) is due to the decreased NSC proliferation but not apoptosis.

**Fig. 2:**
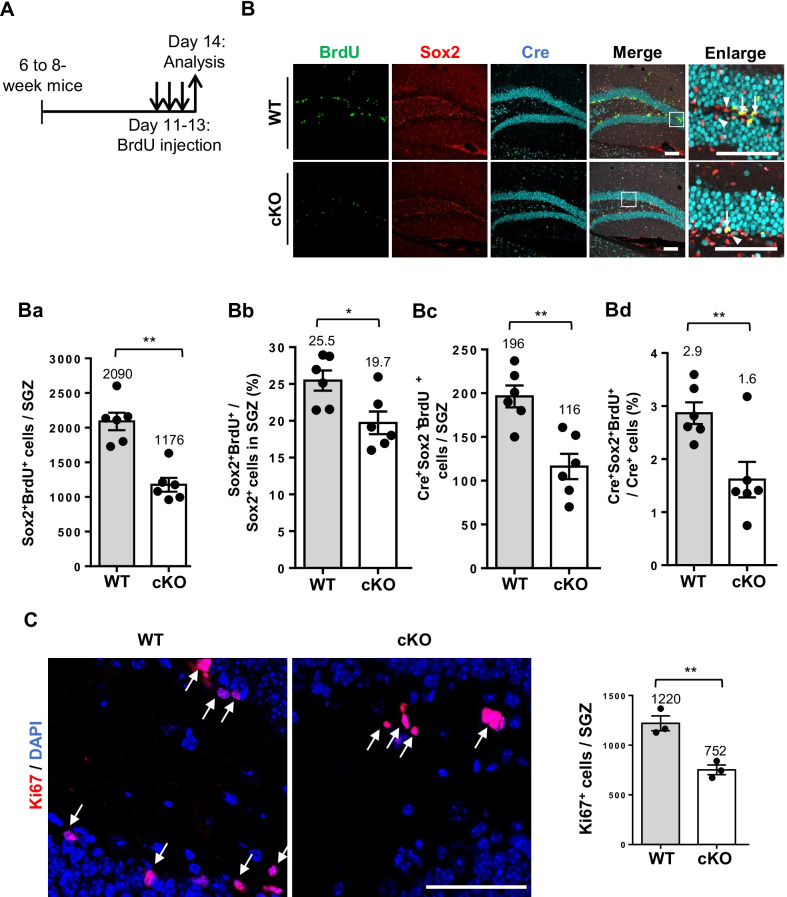
BCAS2 cKO mice show reduced number and proliferation of Sox2^+^ NSCs. **A** The scheme for Sox2^+^NSC proliferation analysis. BrdU administration on day 11 for 3 days in mice at age 6 weeks. **B** Representative images of Sox2^+^BrdU^+^Cre^+^ cells in SGZ by confocal microscopy. Scale bar 100 μm. Quantification of **Ba** Sox2^+^BrdU^+^ cells, **Bb** proportion of Sox2^+^BrdU^+^/Sox2^+^ cells, **Bc** Cre^+^Sox2^+^BrdU^+^ cells, and **Bd** proportion of Cre^+^Sox2^+^BrdU^+^/Cre^+^ cells in SGZ. Arrows: Cre^+^Sox2^+^BrdU^+^ Arrowheads: Sox2^+^. **C** Representative images of Ki67^+^ expression in dentate gyrus (left). Scale bar: 100 μm. Arrows: Ki67^+^. Right: quantification of Ki67^+^ cells in SGZ. Data are mean ± SEM by Student’s *t* test. *P < 0.05; **P < 0.01

To further examine the role of BCAS2 in Sox2^+^ NSC proliferation, we administered BrdU to BCAS2 cKO mice at age 6–8 weeks to label dividing cells (Fig. 2A) [29]. Total of four pieces from every 12th section of 40-μm vibratome hippocampus sections were stained with anti-BrdU and anti-Sox2 for proliferative NSCs labeling. The co-localization of Sox2 and BrdU was captured by the confocal microscope and showed the Z-stack image of Sox2^+^BrdU^+^-expressing cells in WT and BCAS2 cKO mice (Fig. 2B). The stereological analysis [21] described in materials and methods was used to count Sox2^+^BrdU^+^-expressing cells. The number of Sox2^+^BrdU^+^ cells was significantly lower in the SGZ of cKO than WT mice (Fig. 2Ba; 1176 vs. 2090 cells, *P* < 0.01), as was the proportion of Sox2^+^BrdU^+^/Sox2^+^ cells (Fig. 2Bb; 19.7% vs. 25.5%, *P* < 0.05). Different populations of cells coexist in the hippocampus, including quiescent NSCs, amplifying neural progenitors, early differentiating neuroblasts, and maturing granule cells [17]. To precisely examine the direct effect of BCAS2 deletion (Cre^+^) on NSC proliferation, we detected the number of Cre^+^Sox2^+^BrdU^+^ cells, which was lower in the SGZ of cKO than WT mice (Fig. 2Bc; 116 vs. 196, *P* < 0.01), as was the proportion of Cre^+^Sox2^+^BrdU^+^/Cre^+^ cells (Fig. 2Bd; 1.6% vs. 2.9%, *P* < 0.01). To further confirm the reduced BCAS2 level in impaired cell proliferation in the DG, we used Ki67 as a cell proliferative marker. Ki67 is a non-histone nuclear protein widely used for a biomarker for cell proliferation. Ki67 protein can express in all phases of cell cycle, except G_0_ [30]. IFA staining of 8-week-old cKO mice coronal brain sections showed a notable reduction in number of Ki67^+^ cells in the SGZ of cKO versus WT mice (Fig. 2C; 752 vs. 1220 cells, *P* < 0.01). Thus, in cells lacking BCAS2, BCAS2 co-localizing with Sox2 and depletion of BCAS2 level reduces the amount and proliferation of NSCs.

**Fig. 3:**
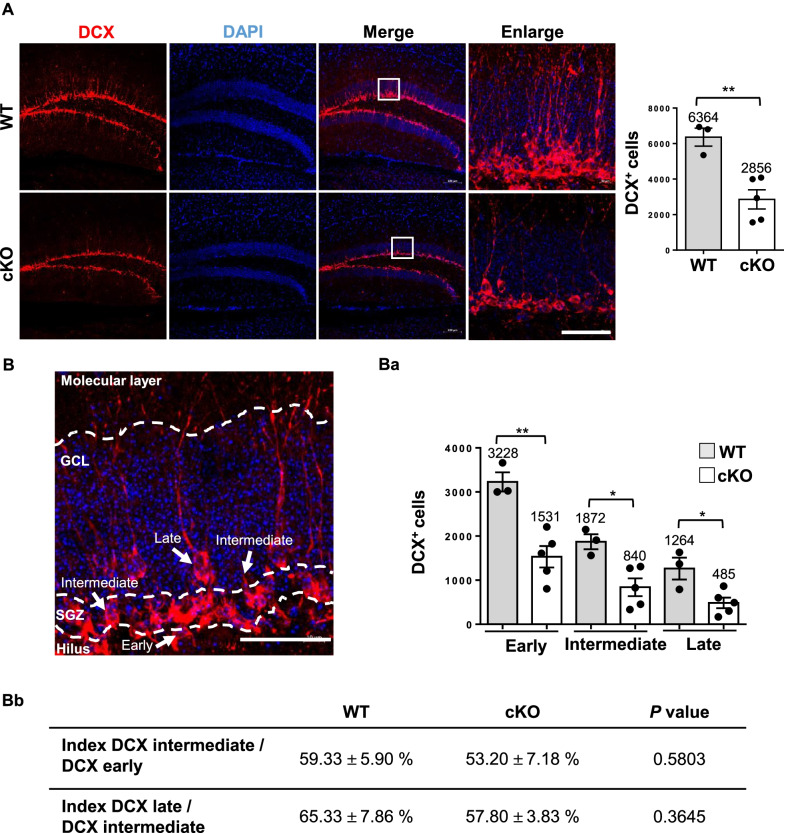
Reduced number of DCX^+^ immature neurons in hippocampus of BCAS2 cKO mice. **A** Left: representative images of DCX expression in hippocampal dentate gyrus. The 40-μm vibratome sections of 8-week-old WT and BCAS2 cKO mice underwent IFA staining for DCX. Scale bar: 50 μm. Right: quantification of DCX^+^ cells. **B** The definition of early, intermediate, and late phase of DCX^+^. DCX^+^ cells distinguished at different stages based on their morphology. Early-stage neuroblasts have cell bodies laying on the SGZ horizontally without dendritic processes. Intermediate-stage newborn neurons have cell bodies vertical and migrate to the granular cell layer (GCL), while exhibiting one or more processes confined to the GCL. Late-stage immature neurons also locate cell bodies at the GCL, but their dendrites have already extended into the molecular layer. **Ba** Quantification of different stages of DCX^+^ cells in dentate gyrus and **Bb** ratio of intermediate to early stage and late to intermediate stage. Data are mean ± SEM by Student’s *t* test. *P < 0.05; **P < 0.01

DCX^+^ represents immature neurons, which will differentiate into newborn neurons during neurogenesis. We used DCX^+^ IFA staining to examine this observation. The 8-week-old cKO mice showed reduced number of DCX^+^ cells as well as shorter dendrites versus WT mice (Fig. 3A). According to the location of cell bodies in DCX^+^ cells, cKO mice showed significantly decreased DCX^+^ neurons as compared with WT mice (Fig. 3A, right panel; 2856 vs. 6364 cells, *P* < 0.01). We identified DCX^+^ cells based on different development stages [31]. Early-stage neuroblasts had cell bodies laying on the SGZ horizontally without dendritic processes. Intermediate-stage newborn neurons had vertical cell bodies that migrated into the granule cell layer (GCL) while exhibiting one or more processes confined to the GCL. Late-stage immature neurons also located cell bodies at the GCL, but their dendrites already extended into the molecular layer (Fig. 3B). Accordingly, the number of DCX^+^ cells was significantly less in cKO than in WT mice in the early stage (Fig. 3Ba; 1531 vs. 3228 cells, *P* < 0.01), intermediate stage (840 vs. 1872 cells, *P* < 0.05), and late stage (485 vs. 1264 cells, *P* < 0.05). Hence, every stage of DCX^+^ in cKO mice was lower than that for WT mice. To determine whether the loss of DCX^+^ cells in cKO was specifically derived from the initial stage or due to immature differentiation. Hence, the index of intermediate/early was calculated and showed no significant difference between WT and cKO (intermediate/early, WT vs. cKO: 59.33% vs. 53.20%, *P* = 0.5803); neither the index of late/intermediate (65.33% vs. 57.80%, *P* = 0.3645) (Fig. 3Bb). It indicated that the depletion of BCAS2 reduced the total DCX^+^ cells but did not affect DCX^+^ cells differentiation from early stage to intermediate or intermediate to late stages. Thus, BCAS2 knockout affects the reduced number of DCX^+^ immature neurons during adult neurogenesis.

Previously BCAS2 cKO mice showed poor dendrite formation [5], so we assumed that BCAS2 also plays a role in neuron maturation. BrdU was administered at day 0 for four consecutive days, and at day 28, immunostaining was performed (Additional file 1: Figure S1B). The number of newborn neuron cells (BrdU^+^NeuN^+^) was significantly lower in cKO than in WT mice (Additional file 1: Figure S1C, Ca; 676 vs. 1106 cells, *P* < 0.05), as was the proportion of BrdU^+^NeuN^+^ to total BrdU^+^ cells (Additional file 1: Figure S1Cb; 68.6% vs. 73.6%, *P* < 0.05). Hence, BCAS2 knockout reduced the number of newborn neuron cells available for maturation, so BCAS2 might be involved in neuron differentiation/maturation. Loss of BCAS2 expression in the forebrain reduced proliferating NSCs and DCX^+^ immature neurons differentiating into mature neuron during adult neurogenesis, and thus, in this study, we focused on Sox2^+^ and DCX^+^ assay for adult neurogenesis.

### Reduced adult neurogenesis in hippocampus of mice with BCAS2 knockdown

**Fig. 4:**
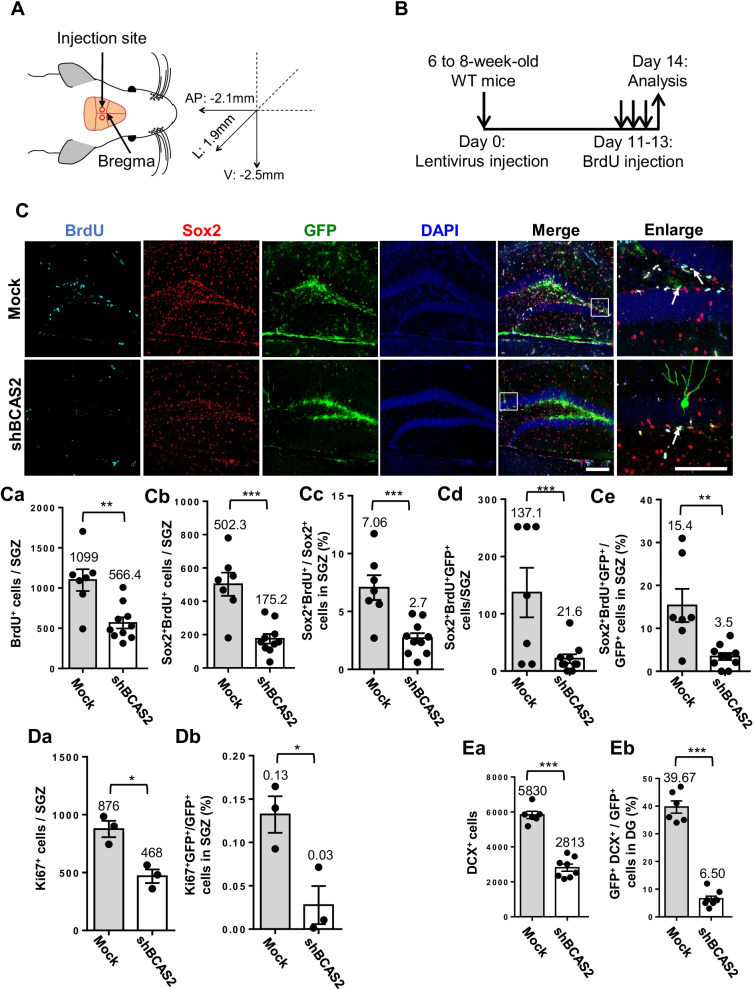
Reduced adult neurogenesis in hippocampus in BCAS2-knockdown mice. **A** Stereotactic surgery delivering lentivirus into the hippocampal dentate gyrus; the spatial coordinates relative to bregma were for anterior and posterior (AP) = − 2.10 mm; lateral (L) = 1.9 mm; ventral (V) = 3.2 mm. **B** Diagram of lentivirus-shBCAS2 intracranial injection (mock: lentivirus-GFP as control) at age 6–8 weeks, day 11 for intraperitoneal BrdU administration for 3 consecutive days and day 14 for analysis. **C** Representative images of Sox2^+^BrdU^+^GFP^+^ cells; Arrows: Sox2^+^BrdU^+^GFP^+^. Scale bar: 100 μm. Approximately 6.7% GFP^+^ cells were located in Sox2^+^-expressing NSCs. Quantification of **Ca** BrdU^+^, **Cb** Sox2^+^BrdU^+^, **Cc** Sox2^+^BrdU^+^/Sox2^+^, **Cd** Sox2^+^BrdU^+^GFP^+^, and **Ce** Sox2^+^BrdU^+^GFP^+^/GFP^+^ cells in SGZ. **D** Ki67^+^ cells in SGZ in BCAS2-knockdown mice (shBCAS2) compared to controls (Mock). Quantification of **Da** Ki67^+^ and **Db** GFP^+^Ki67^+^/GFP^+^ cells and **E** DCX^+^ immature neurons in hippocampal dentate gyrus. Quantification of **Ea** DCX^+^ immature neurons and **Eb** percentage of GFP^+^DCX^+^/GFP^+^ cells. Data are mean ± SEM by Student’s *t* test. *P < 0.05; **P < 0.01; ***P < 0.001

The number of Sox2^+^ NSCs was lower in BCAS2 cKO than in WT mice (Fig. 2) because of deleted BCAS2: the proportion of CaMKIIα expression in Sox2^+^ NSCs was only 3.3% in the DG (Additional file 1: Figure S2). Low CaMKIIα-iCre expression must be avoided in Sox2^+^ cells because interpreting BCAS2-regulating adult neurogenesis is difficult. Thus, we used a stereotaxic intracranial injection of lentivirus-shBCAS2 to knock down BCAS2 in the hippocampus of WT mice (*N* = 7) and mock using lentivirus-GFP (*N* = 7); the spatial injection was relative to the bregma as follows: AP =  − 2.10 mm; lateral = 1.9 mm; ventral = 2.5–3.0 mm (Fig. 4A). Then, BrdU injection was conducted (Fig. 4B) and then immunostaining of Sox2, BrdU, and GFP was performed (Fig. 4C). The various parameters were analyzed as follows: the number of proliferating cells (BrdU^+^) in the SGZ was lower in shBCAS2 than mock-treated animals (lentivirus-GFP injection as mock control) (Fig. 4Ca; 566.4 vs. 1099 cells, *P* < 0.01), so knockdown of BCAS2 in the hippocampus could reduce cell proliferation in WT mice. Proliferating Sox2^+^BrdU^+^ number in the SGZ was also significantly decreased in shBCAS2 versus mock-treated animals (Fig. 4Cb; 175.2 vs. 502.3 cells, *P* < 0.005), as was the proportion of Sox2^+^BrdU^+^ cells to Sox2^+^ single-positive cells in the SGZ (Fig. 4Cc; 2.7% vs. 7.06%, *P* < 0.005). We specifically illustrated that the poor NSC proliferation in BCAS2-knockdown cells was directly affected by virus-infected cells (GFP^+^ cells) because the proliferating NSC number (Sox2^+^BrdU^+^GFP^+^) was significantly decreased in shBCAS2 versus mock-treated mice (Fig. 4Cd; 21.60 vs. 137.1 cells, *P* < 0.005), as was the proportion of Sox2^+^BrdU^+^GFP^+^ cells to GFP + single-positive cells (Fig. 4Ce; 3.5% vs. 15.4%, *P* < 0.01). Moreover, the number of Ki67^+^ cells was decreased in shBCAS2 than in mock control mice (Additional file 1: Figure S3); the number of Ki67^+^ cells in the hippocampal DG was significantly lower in shBCAS2 than in mock control mice (Fig. 4Da; 468 vs. 876 cells, *P* < 0.05). The poor cell proliferation was directly affected by virus-infected cells (GFP^+^ cells) because the proportion of Ki67^+^GFP^+^/GFP^+^ cells was significantly lower in shBCAS2- than in mock-treated mice (Fig. 4Db; 0.03% vs. 0.13%, *P* < 0.05), which indicates the autonomous effect of shBCAS2 reducing cell proliferation. We further determined the DCX^+^ cell effect by BCAS2 knockdown (Additional file 1: Figure S4). DCX^+^ cell number was significantly lower in shBCAS2- than in mock-treated mice (Fig. 4Ea; 2813 vs. 5830 cells, *P* < 0.005), as was the proportion of GFP^+^DCX^+^ to GFP^+^ single-positive cells (Fig. 4Eb; 6.50% vs. 39.67%, *P* < 0.005). In summary, BCAS2 knockdown impaired adult neurogenesis by reducing Sox2^+^ proliferation and number of DCX^+^ immature neurons. The reduced neurogenesis with BCAS2 knockdown in the hippocampus is consistent with low adult neurogenesis in BCAS2 cKO mice.

### Association of BCAS2 and β-catenin in the hippocampus

BCAS2 regulates β-catenin expression [5, 9] and BCAS2 could regulate adult neurogenesis (Figs. 2, 4), so we examined the association between BCAS2 and β-catenin in adult neurogenesis. On immunostaining analysis of the expression pattern of β-catenin and BCAS2, β-catenin predominantly expressed in the SGZ and inner GCL of the DG and co-stained with BCAS2 (Additional file 1: Figure S5A), so BCAS2 colocalized with β-catenin in WT mice. The quantitation showed the decreased expression of β-catenin in BCAS2 cKO mice compared to WT (Additional file 1: Figure S5A; 0.42 vs. 1, *P* < 0.05). Additionally, cKO mice-expressing CaMKIIα-iCre showed a high expression of β-catenin in low-level Cre^+^ cells (Additional file 1: Figure S5A, arrow) and a low expression of β-catenin in high-level Cre^+^ cells (Additional file 1: Figure S5A, arrowheads). These results in mice at age 6 weeks were consistent with our previous results in mice at age 12 weeks [5]. Previously, we had characterized the splicing of β-catenin in brain hippocampal tissues, the RNAs from the hippocampal tissues of WT and BCAS2 cKO, show an accumulation of pre-mRNA and a reduced mRNA level in BCAS2 cKO mice compared with WT, indicating that BCAS2 can regulate the β-catenin pre-mRNA splicing in the hippocampus [5]. Here, to further confirm that BCAS2-regulating adult neurogenesis through β-catenin, we analyzed c-Myc, a downstream targeted gene of β-catenin, that regulates neural stem cell quiescence and activation [32, 33]. We performed c-Myc staining as an indicator of nuclear β-catenin. The BCAS2 cKO mice showed the lower expression of c-Myc^+^ cells compared to WT mice in SGZ (Additional file 1: Figure S5B; 173.3 vs. 248.0, *P* < 0.05), which was in consistent with the downregulation of β-catenin expression in BCAS2 cKO mice (Additional file 1: Figure S5A). Furthermore, western blot analysis of hippocampal lysates confirmed the decreased protein expression of β-catenin and c-Myc in the hippocampus of cKO mice as compared with WT mice at both 6 and 12 weeks (Additional file 1: Figure S5C, left panel). The quantitative data showed that the expression of β-catenin was lower in BCAS2 cKO mice compared to WT mice (Additional file 1: Figure S5C; middle panel 6 week; 0.47 vs. 1, *P* < 0.05, and 12 weeks; 0.39 vs. 1, *P* < 0.05). Similarly, the quantitation showed that the expression of c-Myc was lower in BCAS2 cKO mice compared to WT mice (Additional file 1: Suppl. Figure 5C; right panel 6 weeks; 0.37 vs. 1, *P* < 0.01, and 12 weeks; 0.47 vs. 1, *P* < 0.01), indicating that low BCAS2 was correlated with low β-catenin and c-Myc expression. Hence, BCAS2 regulates adult neurogenesis and neuron maturation in the hippocampus via β-catenin.

**Fig. 5:**
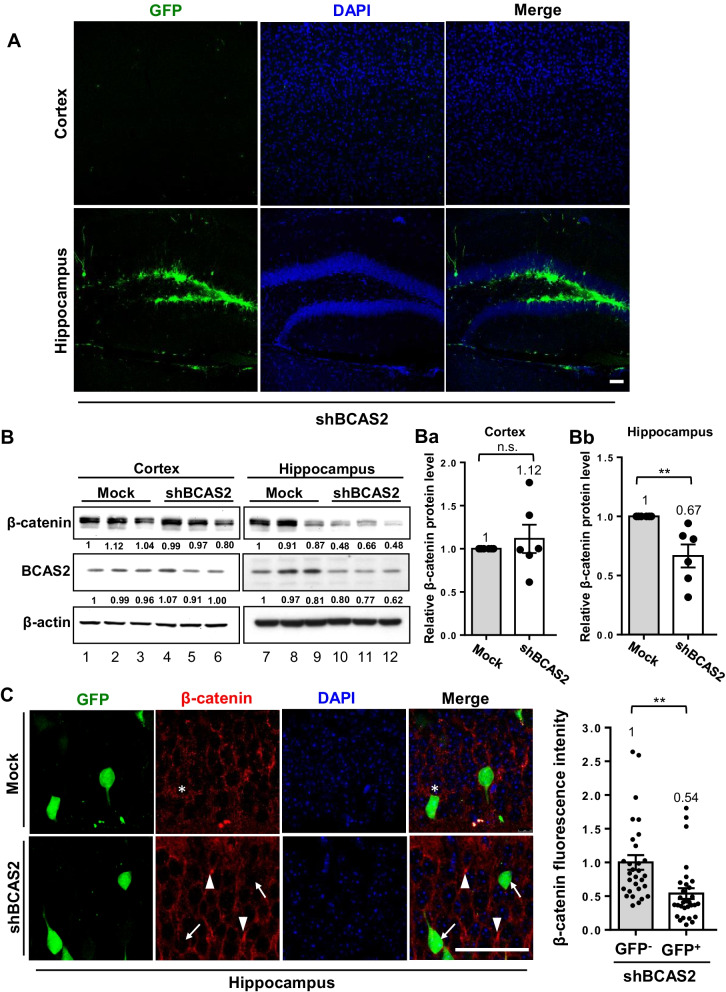
Reduced β-catenin expression in hippocampus of BCAS2-knockdown mice. **A** Representative images of GFP expression in cortex and hippocampus at 2 weeks after lentivirus injection in hippocampal dentate gyrus (shown in Fig. 4A). Scale bar: 50 μm. **B** Western blot analysis of BCAS2 and β-catenin protein levels with lysates from cortex and hippocampus under mock and shBCAS2 treatment. Quantification of relative β-catenin protein level in cortex (**Ba**) and hippocampus (**Bb**). N = 6 for each group. **C** Representative staining of GFP^+^ and β-catenin. Arrows: GFP^+^ cells (BCAS2-knockdown cells) showing low expression of β-catenin. Arrowheads: GFP^−^ cells (non-infected cells) showing high expression of β-catenin. Scale bar: 50 μm. Right: quantification of β-catenin expression intensity in GFP^+^ and GFP^−^ cells in shBCAS2 mice. Ten single cells for each mouse were counted from three separate experiments. Data are mean ± SEM by Student’s *t* test. **P < 0.01, *ns* not significant

We further examined β-catenin expression in the shBCAS2-knockdown mouse hippocampus. No GFP-positive cells could be seen in the cortex, but many were found in the hippocampus (Fig. 5A). Western blot analysis revealed significantly low β-catenin protein in the hippocampus of shBCAS2 versus mock-treated mice (Fig. 5Bb; 0.67 vs. 1, *P* < 0.01) but not in the cortex (Fig. 5Ba; 1.12 vs. 1, *P* > 0.05). It indicated lentivirus-shBCAS2 injection precisely at the hippocampus. To specifically address BCAS2 knockdown correlating with less β-catenin expression, we measured the β-catenin expression in lentivirus-shBCAS2-GFP-infected cells (shBCAS2 group). The GFP^+^ cell meaning shBCAS2 infected cell; the GFP^+^ cells showed low β-catenin expression (arrows), and GFP^−^ cells (meaning non-infected cells) relatively showed high β-catenin expression (arrowheads) (Fig. 5C; 30 cells for each). Quantitation analysis revealed higher β-catenin expression of GFP^−^ cells than GFP^+^ in shBCAS2-injected mice (Fig. 5C right panel; 0.54 vs. 1, *P* < 0.01). The reduced β-catenin fluorescence intensity in GFP^+^ cells compared to GFP^−^ cells was measured precisely from lentivirus-shBCAS2-infected cells (Fig. 5C, right panel); western blot analysis showed the reduced β-catenin expression in lentivirus-shBCAS2-infected hippocampus compared to mock-infected hippocampus was counted from the whole hippocampus tissue extracts than those contained lentivirus-infected cells and non-infected cells (Fig. 5Bb). That may be the reason for the decreased β-catenin expression by western blot (Fig. 5Bb) showed 33% decrease and immunofluorescence stain 46% reduction (Fig. 5C). Thus, BCAS2 might regulate adult hippocampal neurogenesis via β-catenin.

BCAS2 regulates Delta pre-mRNA splicing in Drosophila [34] and Delta-like protein 1 (Dll1) pre-mRNA splicing in MCF7 cells [5]. The Dll1 is the ligand of Notch, and the Notch signaling is required for neural stem cell maintenance and activation [35, 36]. To determine whether BCAS2 regulates Dll1 pre-mRNA splicing in mouse hippocampus, we extracted the RNA from the hippocampus of WT and BCAS2 cKO mice and analyzed the expression of Dll1 mature mRNA and pre-mRNA through quantitative RT-PCR. The results showed no significant difference of Delta-mature mRNA and pre-mRNA expression between WT and BCAS2 cKO mice hippocampus (Additional file 1: Figure S5D; 0.95 vs. 0.82 in Dll1 mRNA, *P* = 0.14 and 1.04 vs. 1.09 in Dll1 pre-mRNA, *P* = 0.52). Moreover, the expression level of Hes1 by IFA, the downstream target of Delta–Notch signaling, in hippocampus DG of BCASC2 cKO mice was similar as WT mice (Additional file 1: Figure S5E; 312 vs. 336, *P* = 0.72). Hence, BCAS2-regulating adult neurogenesis is not through Delta–Notch signaling.

### AAV-DJ8 virus-targeted cells

**Fig. 6:**
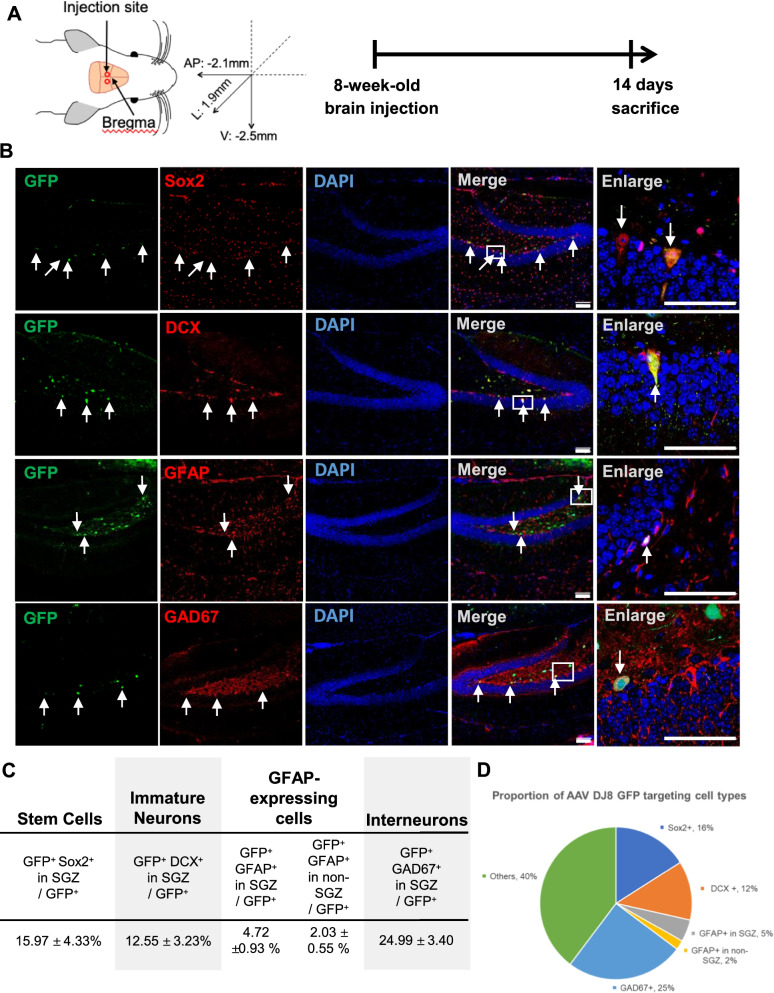
Intracranial injection of AAV-DJ8-targeted cells. **A** Diagram of AAV intracranial injection strategy. Abbreviations as in Fig. 4. WT mice at age 8 weeks were injected with AAV-DJ8-GFP and killed 2 weeks after injection. **B** Representative images of AAV-DJ8-GFP-targeted cells. The cells in hippocampal dentate gyrus include stem cells (Sox2), immature neurons (DCX), astrocytes (GFAP) and interneurons (GAD67). The 40-μm vibratome coronal brain sections of mice underwent IFA staining. Arrows: indicated cells. Scale bar: 100 μm. **C** Proportion of GFP^+^Sox2^+^, GFP^+^DCX^+^, GFP^+^GFAP^+^ cells (in SGZ or not in SGZ) and GFP^+^GAD67^+^ to total GFP^+^ cells in dentate gyrus: 3 sections per mouse from 3 individual mice. **D** The pie chart of AAV-DJ8-GFP-targeted cells. Data are mean ± SEM by Student’s *t* test

Mice with BCAS2 cKO (Fig. 2) and intracranial BCAS2 knockdown (Fig. 4) showed low adult neurogenesis via β-catenin; thus, BCAS2 participates in regulating adult hippocampal neurogenesis. We wondered whether exogenous BCAS2 could rescue Sox2^+^ NSC proliferation and DCX^+^ cells in BCAS2 cKO. First, we wondered about the targeted cells of AAV intracranial injection. AAV can cross the blood–brain barrier and target precise neurons and astrocytes via CMV-promoter-driven gene expression [37]. AAV-DJ8-GFP virus was intracranially injected in the DG of 8-week-old WT mice (coordinates relative to the bregma: anterior and posterior: − 2.1 mm; lateral: ± 1.9 mm; ventral: − 2.5 mm) (Fig. 6A). Two weeks after injection, 40-µm coronal vibratome sections underwent IFA staining. AAV-DJ8 virus injection could efficiently infect several types of cells including NSCs, astrocytes, immature neuron cells, and interneurons (Fig. 6B). The proportion of GFP^+^Sox2^+^, GFP^+^DCX^+^, GFP^+^GFAP^+^, and GFP^+^GAD67^+^ cells to total GFP^+^ cells targeted by AAV-DJ8-GFP in the DG was 15.97 ± 4.33%, 12.55 ± 3.23%, 6.73 ± 1.45%, and 24.99 ± 3.40%, respectively. Because GFAP^+^ cells in the SGZ are potentially directed into stem cells, GFP^+^GFAP^+^ cells in and not in the SGZ were counted separately (Fig. 6C; 4.72% vs. 2.03%). The pie chart of the AAV-DJ8-GFP target cell distribution showed that AAV-DJ8-GFP could infect astrocytic cells (GFP^+^GFAP^+^ not in SGZ) and interneuron cells (Fig. 6D). Thus, AAV-DJ8 GFP could efficiently target NSCs and also non-neuronal cells, such as astrocytes and interneurons.

### AAV-BCAS2 can rescue the neurogenesis of BCAS2 cKO via β-catenin

**Fig. 7:**
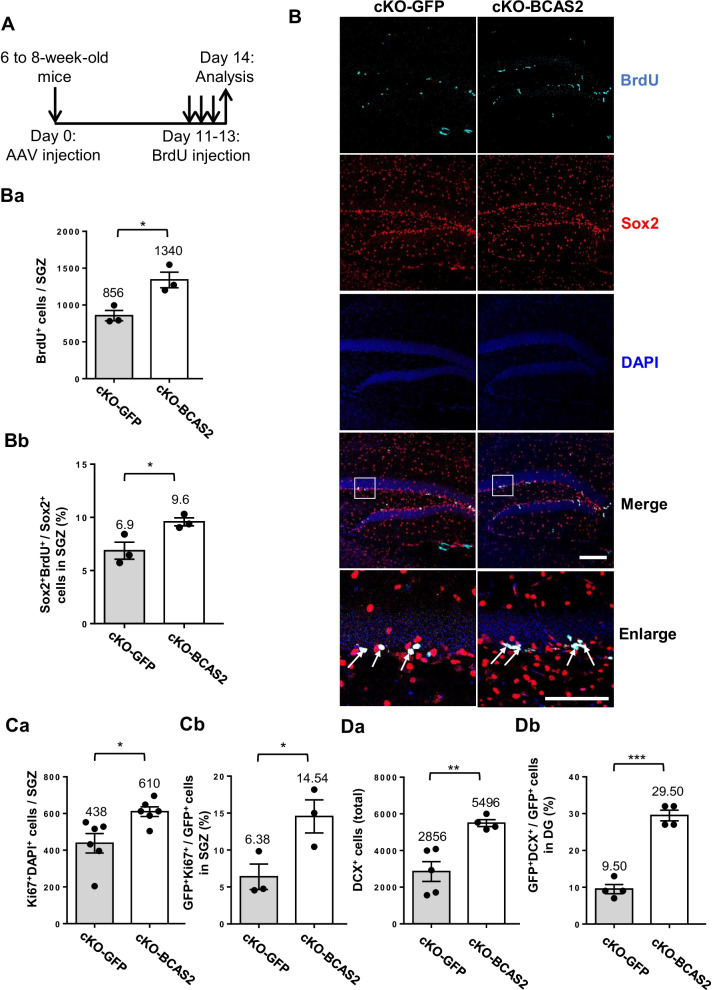
Intracranial injection of AAV-BCAS2 can rescue neurogenesis in hippocampus of BCAS2 cKO mice. **A** Diagram of AAV intracranial injection strategy on days 0 and 11 for BrdU intraperitoneal administration for three consecutive days, and day 14 for analysis. **B** Representative images of BrdU^+^Sox2^+^ cells. Arrows: BrdU^+^Sox2^+^ cells. Scale bar: 100 μm. Quantification of **Ba** BrdU^+^ cells and **Bb** proportion of Sox2^+^BrdU^+^/Sox2^+^ cells in SGZ. Data are mean ± SEM by Student’s t test. **C** Ki67^+^ cells. Quantification of **Ca** Ki67^+^ and **Cb** GFP^+^Ki67^+^/GFP^+^ cells. **D** DCX^+^ immature neurons in hippocampal dentate gyrus. Quantification of **Da** DCX^+^ and **Db** GFP^+^DCX^+^/GFP^+^ cells. Data are mean ± SEM by Student’s *t* test, *P < 0.05; **P < 0.01; ***P < 0.005

Recombinant AAV-DJ8-BCAS2 was generated, and GFP was tagged at the 3’ end. AAV-GFP or AAV-BCAS2 (1 × 10^11^ gc/ml) was stereotaxically intracranially injected into BCAS2 cKO mice at age 6-to-8 weeks. The expression of AAV-GFP or AAV-BCAS2 was both mainly in the hippocampus DG in which the proliferative NSCs were labeled by BrdU; the GFP^+^BrdU^+^ cells were indicated by arrows (Additional file 1: Figure S6A). At 14 days after injection (Fig. 7A), the number of BrdU^+^-proliferating cells in the SGZ was higher in BCAS2 cKO than in GFP-treated cKO mice (Fig. 7Ba; 1340 vs. 786 cells, *P* < 0.05), as was the proportion of Sox2^+^BrdU^+^ to Sox2^+^ single-positive cells (increased 63.6%) (Fig. 7Bb; 9.6% vs. 6.1%, *P* < 0.05). We examined whether AAV-BCAS2 could restore the cell proliferation in BCAS2 cKO mice by using Ki67 (Additional file 1: Figure S6B); the number of Ki67^+^ cells in the SGZ significant increased after AAV-BCAS2 treatment versus the GFP control (Fig. 7Ca; 610 vs. 438 cells, *P* < 0.05). The proportion of GFP^+^Ki67^+^/GFP^+^ cells indicated an autonomous effect by AAV-BCAS2 restoring cell proliferation (Fig. 7Cb; cKO-BCAS2 14.54% vs. cKO-GFP 6.38%, *P* < 0.05). Hence, the forced expression of BCAS2 in the hippocampus of BCAS2 cKO mice could rescue the proliferation of Sox2^+^ NSCs. Moreover, AAV-BCAS2 restored the number of DCX^+^ immature neurons in BCAS2 cKO mice (Additional file 1: Figure S7 and Fig. 7Da; 5496 vs. 2856 cells, *P* < 0.01). The proportion of GFP^+^DCX^+^/GFP^+^ cells was higher with BCAS2 than GFP cKO (Fig. 7Db; 29.50% vs. 9.50%, *P* < 0.005), which indicates the autonomous effect of BCAS2 increasing the number of DCX^+^ immature neurons. Hence, AAV-BCAS2 could restore poor neurogenesis in BCAS2 cKO mice caused by depletion of BCAS2, which shows the therapeutic potential of intracranially injected AAV-BCAS2.

On the other hand, The GFP^+^ cells in Fig. 4 were the lentivirus-GFP and lentivirus-GFP-shBCAS2 infected cells, and the GFP^+^ cells in Additional file 1: Figure S6 were the AAV-GFP- and AAV-GFP-BCAS2-infected cells. Hence, the virus vector we used in Fig. 4 (lentivirus) and in Additional file 1: Figure S6 (adeno-associated virus, AAV) was different. Hence, the infection rate would be different between lentivirus and AAV to result in the different GFP expression patterns between Fig. 4 and Additional file 1: Figure S6.

**Fig. 8:**
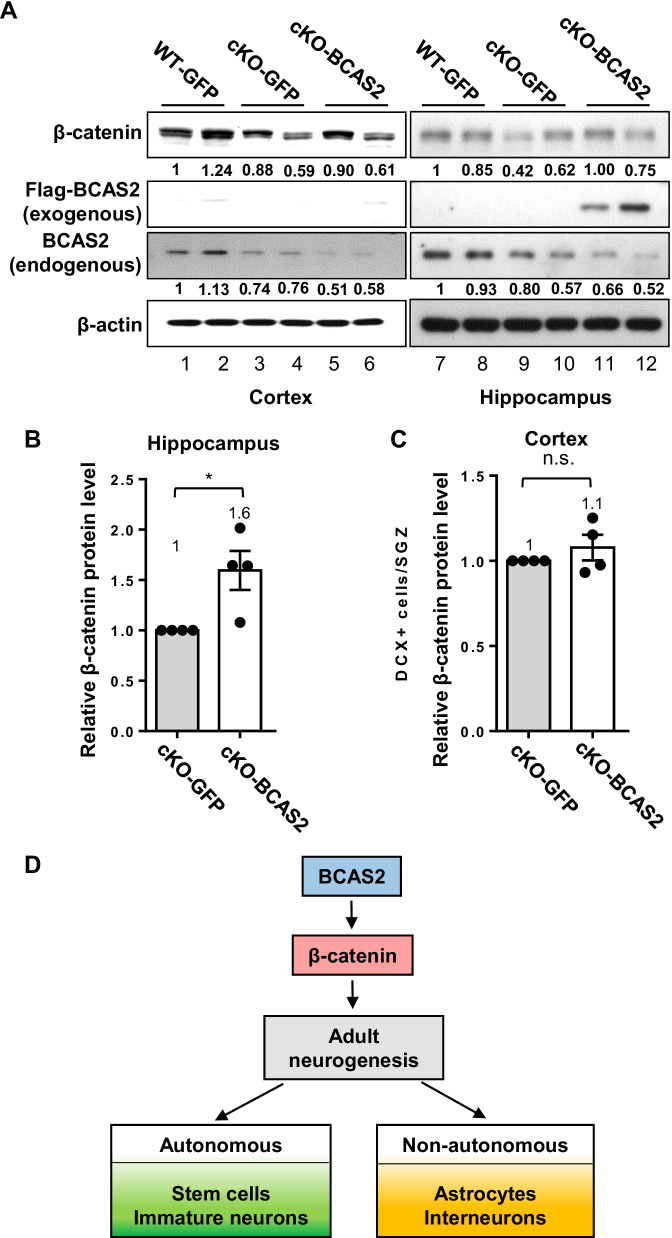
Intracranial injection of AAV-BCAS2 can restore β-catenin level in hippocampus of BCAS2 cKO mice. **A** Western blot analysis of exogenous BCAS2-GFP and endogenous BCAS2 protein levels in the cortex and hippocampus of cKO-BCAS2 mice after AAV-BCAS2 and AAV-GFP treatment. The endogenous BCAS2 level was decreased in cKO mice as described previously [5]. Quantification of β-catenin protein level in **B** hippocampus and **C** cortex. **D** Working model of BCAS2 inducing adult neurogenesis via β-catenin for autonomous (stem cells and immature neurons) and/or non-autonomous effects (astrocytes and interneurons). Data are mean ± SEM. *P < 0.05, *ns* not significant

We further investigated whether the forced expression of BCAS2 in the hippocampus could reverse the downregulated β-catenin level in BCAS2 cKO mice. The gene driven by the CMV promoter can be expressed in the cortex and hippocampus [38, 39]. To specifically deliver AAV-BCAS2 to the hippocampus, we performed the stereotaxic injection in the mouse brain (Fig. 6) to precisely inject the AAV-BCAS2 into DG. Endogenous BCAS2 and β-catenin protein levels were low in both the cortex (Fig. 8A, lanes 3–6) and hippocampus (Fig. 8A, lanes 9–10) of cKO mice, but exogenous BCAS2 protein level was high in the hippocampus (Fig. 8A, lanes 11–12) but not cortex (Fig. 8A, lanes 5–6) of BCAS2-treated cKO mice. The level of β-catenin was increased 1.6-fold in the hippocampus (Fig. 8B, *P *< 0.05) and 1.1-fold in the cortex (Fig. 8C). Thus, BCAS2-regulating adult neurogenesis may at least be via β-catenin, and intracranial virus injection is accurately located in the hippocampus. We propose the working model of BCAS2-inducing adult neurogenesis via β-catenin by autonomous (stem cells and immature neurons) and/or non-autonomous effects (astrocytes and interneurons) (Fig. 8D).

### Intracranial hippocampal injection of AAV-β-catenin in BCAS2 cKO mice could rescue adult neurogenesis

**Fig. 9:**
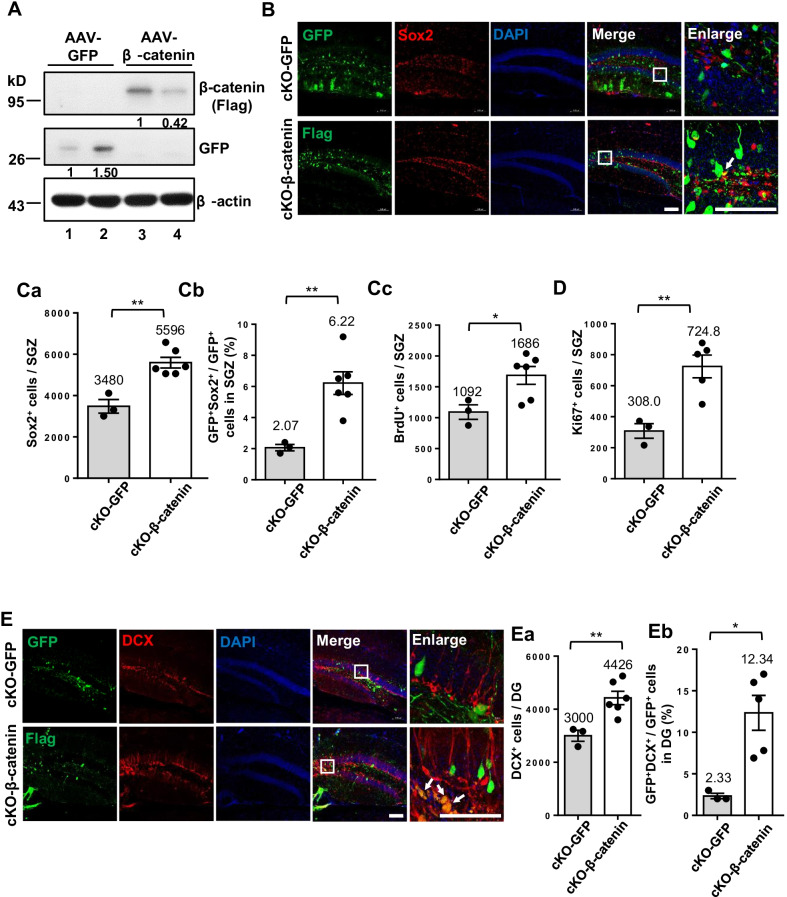
Intracranial hippocampal injection of AAV-β-catenin in BCAS2 cKO mice could rescue adult neurogenesis. **A** Western blot analysis of hippocampus in AAV-β-catenin at 2 weeks after intracranial injection. **B** Representative images of Flag (β-catenin) and Sox2 in hippocampal dentate gyrus (DG) of cKO-GFP and cKO-β-catenin mice. Scale bar: 100 μm. Quantification of **Ca** Sox2^+^, **Cb** GFP^+^ Sox2^+^/GFP^+^ and **Cc** BrdU^+^ cells in SGZ. **D** Quantification of Ki67^+^ cells in SGZ. **E** Representative images of DCX, GFP, and Flag expression. Scale bar: 100 μm. Quantitation of **Ea** DCX^+^/DG and **Eb** GFP^+^DCX^+^ (for cKO-GFP) or Flag^+^ DCX^+^ cells (for AAV-β-catenin) in DG. Data are mean ± SEM by Student’s *t* test, *P < 0.05; **P < 0.01; ***P < 0.005

Reduced expression of β-catenin has been reported in Alzheimer disease (AD) brains; overexpression of wild-type β-catenin in AD glial progenitor cells (GPCs) can restore the expression of β-catenin and neurogenesis in AD, indicating that wild-type β-catenin has a function to activate neurogenesis [40]. Similarly, BCAS2 cKO also showed a decrease of β-catenin expression and neurogenesis (Figs. 1, 2, 3 and Additional file 1: Figure S5). To further verify that BCAS2-regulating neurogenesis is via β-catenin, we tested whether forced expression of wild-type β-catenin in BCAS2 cKO mice could restore neurogenesis. Recombinant AAV-β-catenin (flag tagged at the 3’ end) was generated and purified. Intracranial injection of AAV-β-catenin into brains of mice at age 6–8 weeks involved stereotactic surgery targeting the DG (Fig. 6A). After 2 weeks, western blot analysis showed β-catenin and GFP protein expression in the hippocampus of AAV-β-catenin-treated mice (Fig. 9A, lanes 3–4) and AAV-GFP-treated mice (Fig. 9A, lanes 1–2). IFA also confirmed that GFP (AAV-GFP-treated mice) and Flag (β-catenin) were expressed via AAV-encoded genes (Fig. 9B). To determine whether AAV-β-catenin could restore adult neurogenesis in BCAS2 cKO mice, Sox2 and BrdU levels were analyzed by IFA. The number of Sox2^+^ cells in the SGZ was significantly higher in AAV-β-catenin than in AAV-GFP-treated mice (Fig. 9Ca; 5596 vs. 3480 cells, *P* < 0.01); similarly, the number of BrdU^+^ cells in SGZ was increased in AAV-β-catenin-treated mice (Fig. 9Cc; 1686 vs. 1092 cells, *P* < 0.05). The significant Sox2^+^ increase in Flag-targeted cells (Flag^+^Sox2^+^/Flag^+^) than GFP-targeted cells (GFP^+^Sox2^+^/GFP^+^) indicated the autocrine effect of β-catenin-increased Sox2^+^ stem cells (Fig. 9Cb; 6.22% vs. 2.07%, *P* < 0.01). We further examined whether AAV-β-catenin induced cell proliferation. Ki67 images showed increasing Ki67^+^-proliferating cells in AAV-β-catenin-treated versus AAV-GFP control BCAS2 cKO mice (Additional file 1: Figure S8 and Fig. 9D; 724.8 vs. 308.0 cells, *P* < 0.01). The impaired neuron differentiation in BCAS2 cKO mice was indicated by DCX staining (Fig. 9E): the number of DCX^+^ cells was significantly higher in AAV-β-catenin—than in AAV-GFP-treated BCAS2 cKO mice (Fig. 9Ea; 4426 vs. 3000, *P* < 0.01). The proportion of GFP^+^DCX^+^ to GFP^+^ cells indicated that neuron development was directly promoted by AAV-β-catenin infection (Fig. 9Eb; cKO-β-catenin 12.34% vs. cKO-GFP 2.33%, *P* < 0.05), thus implying its autocrine effect. Taken together, AAV-β-catenin gene therapy could restore the number of Sox2^+^, BrdU^+^, Ki67, and DCX^+^ cells in BCAS2 cKO mice. The forced expression of β-catenin by the AAV vector rescued the proliferation of NSCs and the differentiation of newborn neurons in mice with impaired neurogenesis caused by BCAS2 cKO via an autocrine or paracrine effect.

## Discussion

In this study, we characterized BCAS2-regulating Sox2^+^ NSC proliferation and DCX^+^ immature neurons. BCAS2 co-localized with Sox2 expression in the SGZ of the DG in WT mice (Fig. 1), similar to the co-localization with DCX in our previous report [5]. Postnatal depletion of BCAS2 in the forebrain of mice (BCAS2 cKO) reduced Sox2^+^ NSC cell number, NSC proliferation, number of DCX^+^ immature neurons, and newborn neuron maturation (Figs. 1, 2, 3 and Additional file 1: Figure S1). Stereotaxic intracranial injection of lentivirus-shBCAS2 into the hippocampus, which accurately knocked down BCAS2 expression in the hippocampus, also confirmed BCAS2-regulating adult neurogenesis. Moreover, AAV-BCAS2 gene therapy via intracranial injection into the hippocampus rescued the reduced number and proliferation of Sox2^+^ NSCs and increased the number of DCX^+^ immature neurons in BCAS2 cKO mice. Along with our previous report, here we show that loss of BCAS2 in the adult mouse forebrain impaired hippocampal neurogenesis and decreased the self-renewal ability and proliferation of Sox2^+^ NSCs and DCX^+^ immature neurons, thereby leading to defective dendrite formation [5] and decreased differentiation ability to newborn neurons (Additional file 1: Figure S1). Hence, BCAS2 regulates adult neurogenesis.

Moreover, in this study, serotype-AAV-DJ8-encoded genes administered by intracranial injection could target 16% of stem cells (Sox2^+^), 25% of interneurons and 2% of astrocytes (GFAP^+^ in non-SGZ) (Fig. 6), so the AAV-DJ8 could deliver genes into neuron cells and other non-neuron cells (such as astrocytes and interneurons). AAV gene therapy has been used in many neurodegenerative diseases, such as Parkinson’s disease, Alzheimer disease, and SMA [41–43]. Intracranial or intrathecal injection of AAV serotype 1, 2, 5, 8, and 9 can cross the blood–brain barrier and precisely target the local population of NSCs and non-neural cells such as astrocytes and oligodendrocytes [44]. All of these AAV serotypes can be used for therapeutic gene therapy because the effect of the delivered genes can last a few years in human participants [45]. For example, in an Alzheimer disease mouse model, anti-Aβ single-chain antibody could be delivered into the cortical–hippocampal region by AAV serotype 5 or 9 intracranial injections, with notable decrease in plaque and Aβ peptide level in the cortex and hippocampus without any neurotoxic side effects [46–48]. In Parkinson’s disease, bilateral putaminal infusions of a high or low dose of AAV2-aromatic L-amino acid decarboxylase (AADC) could last at least 4 years, sustaining the expression of AADC, which supports the therapeutic effect of AAV delivery for a few years [49]. In this study, we provide evidence that the administration of AAV-DJ8 serotypes by intracranial injection can target Sox2 stem cells, DCX immature neurons, interneurons and astrocytes.

Injection with AAV-BCAS2 in BCAS2 cKO mice could rescue adult neurogenesis, reaching 57.4% as compared with AAV-GFP injection (Fig. 7Bb; 9.6–6.1/6.1%). Astrocytes or interneurons are targeted with AAV-DJ8-BCAS2 injection, which may suggest a non-autonomous effect on NSCs to enhance adult hippocampal neurogenesis (Fig. 8D). BCAS2 cKO mice showed significantly lower Sox2^+^ NSC proliferation than WT mice (Fig. 2). In the BCAS2 cKO mouse model, deletion of BCAS2 expression was driven by CaMKIIα-iCre. CaMKIIα-iCre expression in Sox2 cells of the SGZ was very low (about 3.3%) (Additional file 1: Figure S2). Similarly, stereotaxic intracranial injection of lentivirus-shBCAS2 with a GFP-tag resulted in approximately 6.7% GFP^+^ cells in Sox2-expressing NSCs (Fig. 4) along with a significant decrease in number of Sox2^+^ NSCs and DCX^+^ immature neurons in the DG of the BCAS2 cKO hippocampus. Taken together, the three systems—BCAS2 cKO in mice, lentivirus BCAS2 knockdown and AAV-BCAS2 gene therapy in BCAS2 cKO mice—provide evidence that BCAS2-regulating adult neurogenesis has autocrine and paracrine effects.

BCAS2 is a core component of the Prp19–CDC5L spliceosome complex that controls RNA splicing [8]. Reportedly, BCAS2 regulates the splicing of spermatogenesis-related genes to regulate spermatogenesis [6]; Delta pre-mRNA splicing to affect Drosophila wing development [4]; and β-catenin to control brain size [5]. As to neurogenesis, Delta–Notch signaling reportedly stimulates the differentiation of neural stem cells in embryonic and adult brains [50, 51]. Hes5, an effector of Delta–Notch signaling regulates the transition timing of neurogenesis in mammalian brain development [52]. On the other hand, β-catenin/TCF signaling is a positive regulator of neurogenesis in the adult hippocampus [53]. BCAS2 regulates both Delta and β-catenin splicing. However, BCAS2 cKO mice in which BCAS2 expression was eliminated in the forebrain show small brains with reduced dentate gyrus (DG) volume, which is resulted from dysregulation of β-catenin pre-mRNA splicing but affects Delta expression [5]. The splicing efficiency of β-catenin by BCAS2 in cKO mice, RNAs from the hippocampal tissues of WT and BCAS2 cKO, shows an accumulation of pre-mRNA and a reduced mRNA level in BCAS2 cKO mice compared with WT, indicating that BCAS2 can regulate the β-catenin pre-mRNA splicing in the hippocampus [5]. Hence, in this study, we would focus on studying BCAS2-regulating neurogenesis via β-catenin. Here, we showed the decreased β-catenin expression in BCAS2 cKO [5] along with low neurogenesis, which showed the decrease of Sox2^+^ NSCs (Fig. 2) and DCX^+^ immature neurons (Fig. 3); similarly, BCAS2-knockdown mice revealed the reduced β-catenin expression (Fig. 5B), low Sox2^+^ NSCs and DCX^+^ immature neurons (Fig. 4). When AAV-BCAS2 gene therapy in BCAS2 cKO could restore β-catenin expression (Fig. 8), and Sox2^+^ NSCs and DCX^+^ immature neurons (Fig. 7). Moreover, forced β-catenin expression in BCAS2 cKO mice could rescue neurogenesis including the increase of Sox2^+^ NSCs and DCX^+^ immature neurons (Fig. 9B, E). Hence, BCAS2 may regulate β-catenin expression [5] for adult neurogenesis. The activation of β-catenin can promote NSC proliferation and increase the neural progenitor pool [15]. With enhanced Wnt signaling, the number of NSCs is increased. Overexpression of β-catenin in mice can increase the neuronal precursor population, with the mouse brain size significantly enlarged [54].

Taken together, BCAS2 regulates Sox2^+^ NSC proliferation and DCX^+^ immature neuron differentiation via β-catenin. Besides BCAS2-regulating β-catenin, our previous report also demonstrated that BCAS2 can regulate *Delta–Notch* signaling [3, 4] and is a negative regulator of p53 [1]. Both factors are associated with adult neurogenesis. How BCAS2 regulates each step of neurogenesis by one signal or many signals remains unknown. In the future, we will further characterize what signals are induced by BCAS2 and how for each neurogenesis step.

## Conclusions

BCAS2 is an essential factor for adult neurogenesis in the hippocampus. The effects of BCAS2 involved in adult neurogenesis via β-catenin may be derived from autonomous effects, from Sox2 stem cells, DCX immature neurons or/and non-autonomous effects from interneurons and astrocytes, etc.

## Supplementary Information

**Additional file 1**. Supplemental Figures.

**Additional file 2**. Supplemental Table.

## Data Availability

Not applicable.

## Abbreviations

NSC: Neural stem cell
WT: Wild type
cKO: Conditional knockout
SGZ: Subgranular zone
AAV: Adeno-associated virus
DG: Dentate gyrus
BrdU: Bromodeoxyuridine
i.p.: Intraperitoneal
IFA: Immunofluorescence analysis
