# Supplementary material for: Breast carcinoma-amplified sequence 2 regulates adult neurogenesis via β-catenin
Source: Stem Cell Res Ther. 2022 Apr 11;13:160. doi: 10.1186/s13287-022-02837-9 (PMC8996563; doi:10.1186/s13287-022-02837-9)

Supplemental figure legends

Supplemental Fig. 1 BCAS2 cKO mice show reduced dentate gyrus (DG) volume and low $NeuN^+$ cells in DG versus WT mice. **A** The DG volume was measured by stereological analysis with ImageJ (NIH), and presented by a bar histogram at age 3 and 6 weeks. The 4-μm paraffin sections of WT and cKO underwent immunohistochemistry assay with anti-NeuN antibody. $NeuN^+$ cell density in the DG was quantified by 50 randomly chosen counting frames (N = 3, counting frame: 25 x 25 μm). Estimated $NeuN^+$ cells in DG were calculated by multiplying the granule cell density with DG volume as described [5]. Data are mean ± SEM by Student's t test, $*P < 0.05$, $**P < 0.01$. **B** Newborn neuron measurement ($NeuN^+$). The scheme of newborn neuron maturation measurement. Daily BrdU injection for 4 constitutive days at age 6 weeks, followed by IFA of newborn neuron maturation with anti-NeuN antibody on day 28. **C** Representative images of $NeuN^+BrdU^+$ by confocal microscopy. Scale bar: 200 μm. Quantification of **Ca** number of $NeuN^+BrdU^+$ cells in DG and **Cb** proportion of $NeuN^+BrdU^+$ to total $BrdU^+$. Data are mean ± SEM by Student's t test. $*P < 0.05$. **D** Representative images of TUNEL positive control. The 40-μm forebrain vibratome sections were incubated with 5 μg/ml DNaseI and underwent FITC-dUTP labeling (green) through TUNEL assay. Negative showed sections without DNaseI incubation. DAPI (blue) is nuclear counter stain. **E** Representative images of TUNEL stain in WT and BCAS2 cKO mice. The hippocampus DG was labeled with FITC-dUTP (green) through TUNEL assay. DAPI (blue) is nuclear counter stain. **F** Representative images of activated caspase-3. The hippocampus DG was stained with anti-activated caspase-3 (green) antibody. DAPI (blue) is nuclear counter stain. Scale bar: 200 μm.

Supplemental Fig. 2 CaMKIIα-Cre expression pattern in $Sox2^+$ NSCs in SGZ of DG. **A** Series of every 12th 40-μm vibratome sections underwent IFA with anti-Sox2 and anti-Cre antibodies. Images were taken by confocal microscopy. $Sox2^+$ cells are indicated by arrowhead and Cre^+ $Sox2^+$ cells by arrows. Scale bar: 100 μm. **B** More than 1000 $Sox2^+$cells were analyzed and Cre was expressed in NSCs by measuring the percentage of Cre^+Sox2^+ cells to Sox2 single-positive cells in the SGZ in WT mice, N = 3.

Supplemental Fig. 3 Reduced number of $Ki67^+$ proliferative cells in SGZ of BCAS2-knockdown mice. Representative images of Ki67 expression in DG in shBCAS2- and mock-treated mice. The 40-μm vibratome coronal brain sections underwent IFA staining for Ki67. Scale bar: 100 μm.

Supplemental Fig. 4 Reduced number of DCX^+ immature neurons in hippocampus of BCAS2-knockdown mice. Representative images of DCX expression in hippocampal DG. Expression of DCX and dendrite formation (arrow) after BCAS2 knockdown in 40-μm vibratome sections after IFA staining. Scale bar: 100 μm.

Supplemental Fig. 5 Loss of BCAS expression reduced β-catenin expression without altering notch signaling in mouse hippocampus. **A** Representative images of BCAS2 and β-catenin. The 10-μm forebrain paraffin sections underwent IFA with mouse anti-β-catenin and rabbit anti-BCAS2 antibodies. WT: β-catenin predominantly expressed in the SGZ and inner granular cell layer of DG and co-stained with BCAS2, indicated by arrow. cKO: IFA analysis of β-catenin expression in Cre-positive cells of cKO. Arrow: low level Cre with high expression β-catenin. Arrowhead: high level Cre with

low β-catenin. Scale bar: 20 μm. Right: Quantification of relative β-catenin IF intensity in SGZ from left panel (N = 4 for each group). **B** Representative images of c-Myc and β-catenin. The 40-μm forebrain vibratome sections underwent IFA with mouse anti-β-catenin (green) and anti-c-Myc (red) antibodies. The nuclear co-localization of c-Myc and β-catenin in SGZ was indicated by arrows. DAPI is nuclear counter stain. Scale bar: 20 μm. Lower: quantitation of the number of c-Myc^+ cells in SGZ from the left panel (N = 3 for each group). **C** Western blot analysis of protein levels of β-catenin and c-Myc in the hippocampus of 3-, 6- and 12-week-old cKO mice. Middle: quantitation of relative β-catenin expression (N=3 for each group). Right: quantitation of relative c-Myc expression (N=3 for each group). **D** Quantitative RT-PCR analysis of the Dll1 mRNA and pre-mRNA expression in the hippocampal tissues of WT and cKO mice (N=4 for each group). **E** Representative images of Hes1 staining in DG. The 40-μm forebrain vibratome sections underwent IFA with mouse anti-Hes1 (red) antibody. The nuclear stain of Hes1 in SGZ was indicated by arrows. DAPI is nuclear counter stain. Scale bar: 20 μm. Right: Quantification of the number of $Hes1^+$ cells in SGZ from the left panel (N = 4 for each group). Data are mean ± SEM by Student's t test. *P < 0.05, **P < 0.01, n.s. not significant.

Supplemental Fig. 6 Intracranial hippocampal injection of AAV-BCAS2 can restore cell proliferation in 8-week-old mice. **A** Representative images of GFP, BCAS2 and BrdU expression in hippocampal DG from AAV-GFP and AAV-BVAS2 treated BCAS2 cKO mice. Arrows: GFP^+BrdU^+ cells. **B** Representative images of Ki67 expression in dentate gyrus in cKO-BCAS2 and cKO-GFP mice. The 40-μm vibratome brain sections underwent IFA staining for Ki67. Scale bar: 100 μm.

Supplemental Fig. 7 Representative images of DCX expression in hippocampal DG of cKO-BCAS2 mice. Increasing expression of DCX and dendrite formation in AAV-BCAS2–injected cKO mice. The 40-μm vibratome sections underwent IFA staining for DCX. Scale bar: 100 μm.

Supplemental Fig. 8 Proliferating cells increased in number in AAV-β-catenin–treated BCAS2 cKO mice. Representative images of Ki67 and Flag (β-catenin) expression in hippocampal DG of cKO-β-catenin mice. Increasing expression of $Ki67^+$ proliferating cells in cKO-β-catenin mice. Scale bar: 100 μm.

Supplemental Figure 1

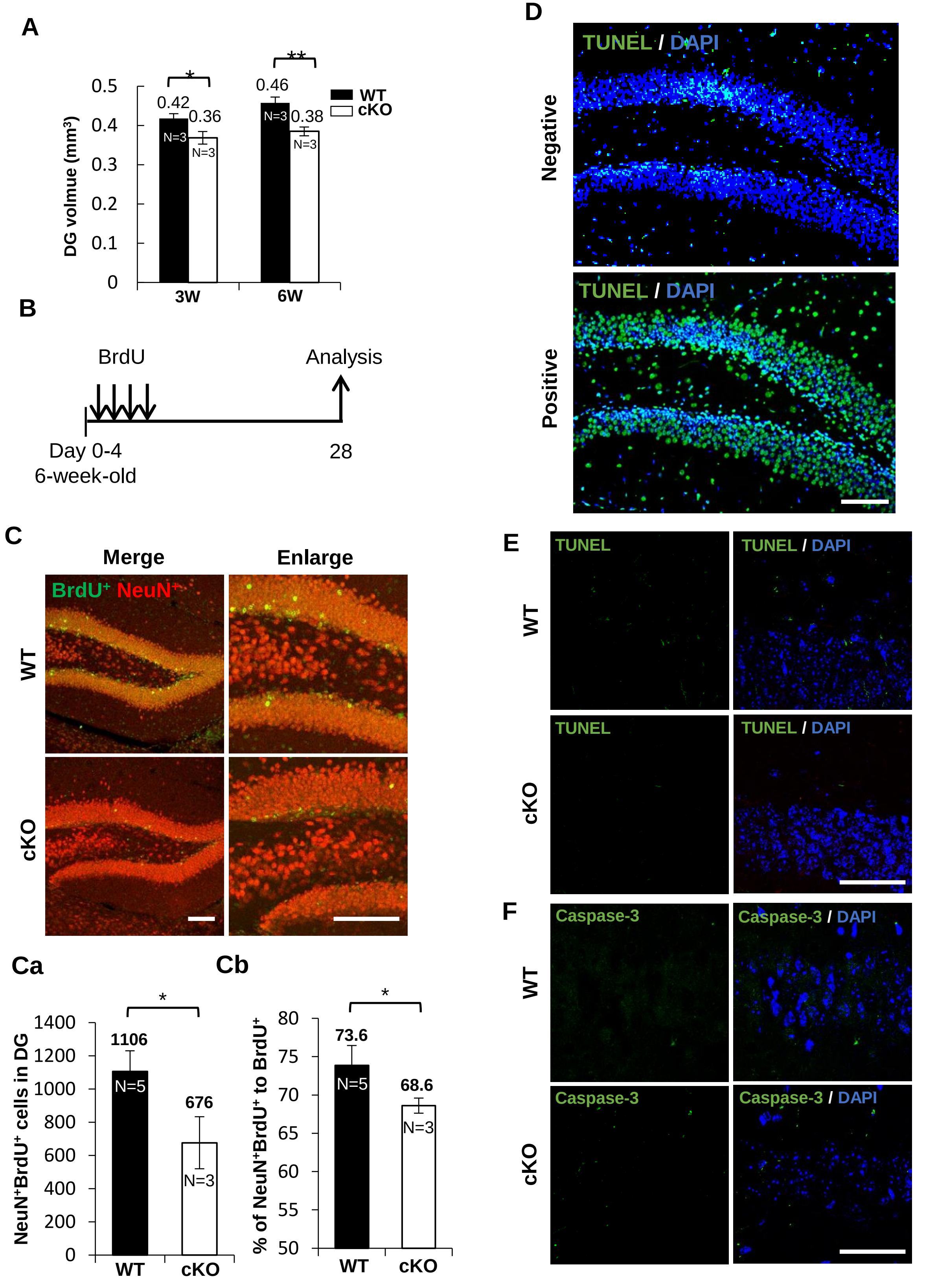

Supplemental Figure 2

A

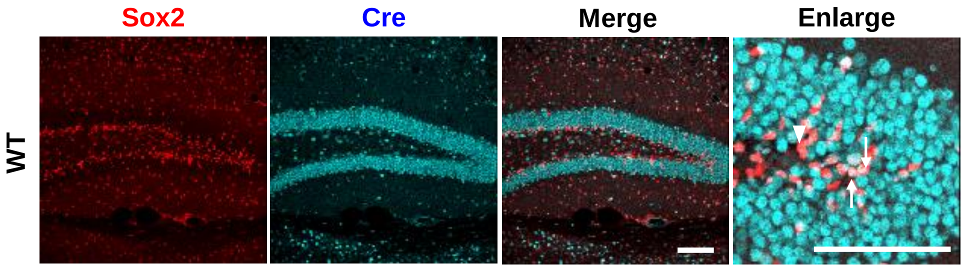

B

	WT Ctrl
Percentage of $Cre^+Sox2^+/Sox2^+$ (±SEM)	3.3% ± 0.25

Supplemental Figure 3

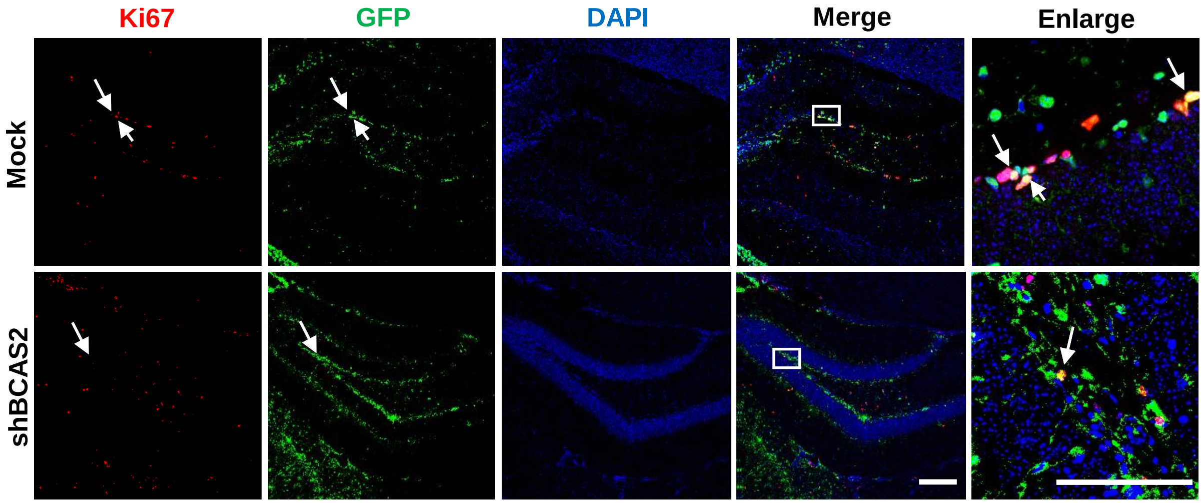

Supplemental Figure 4

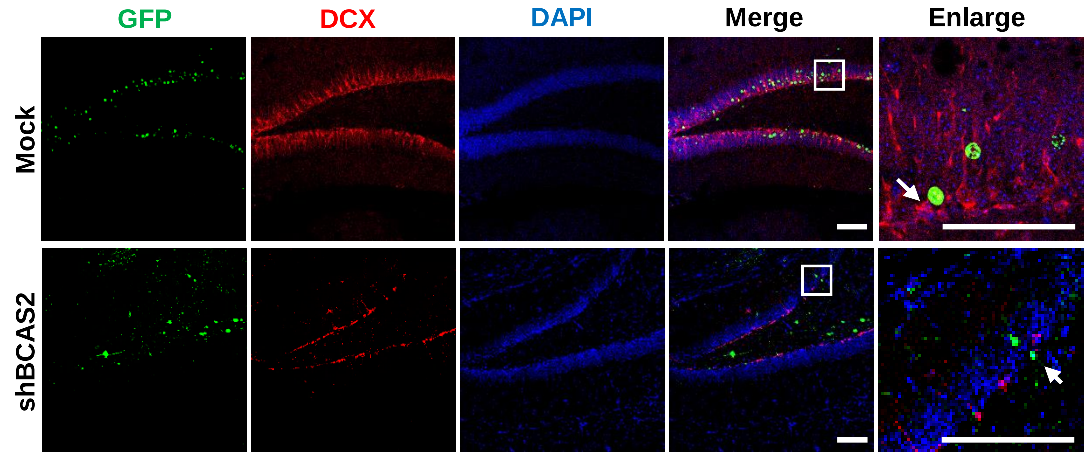

Supplemental Figure 5

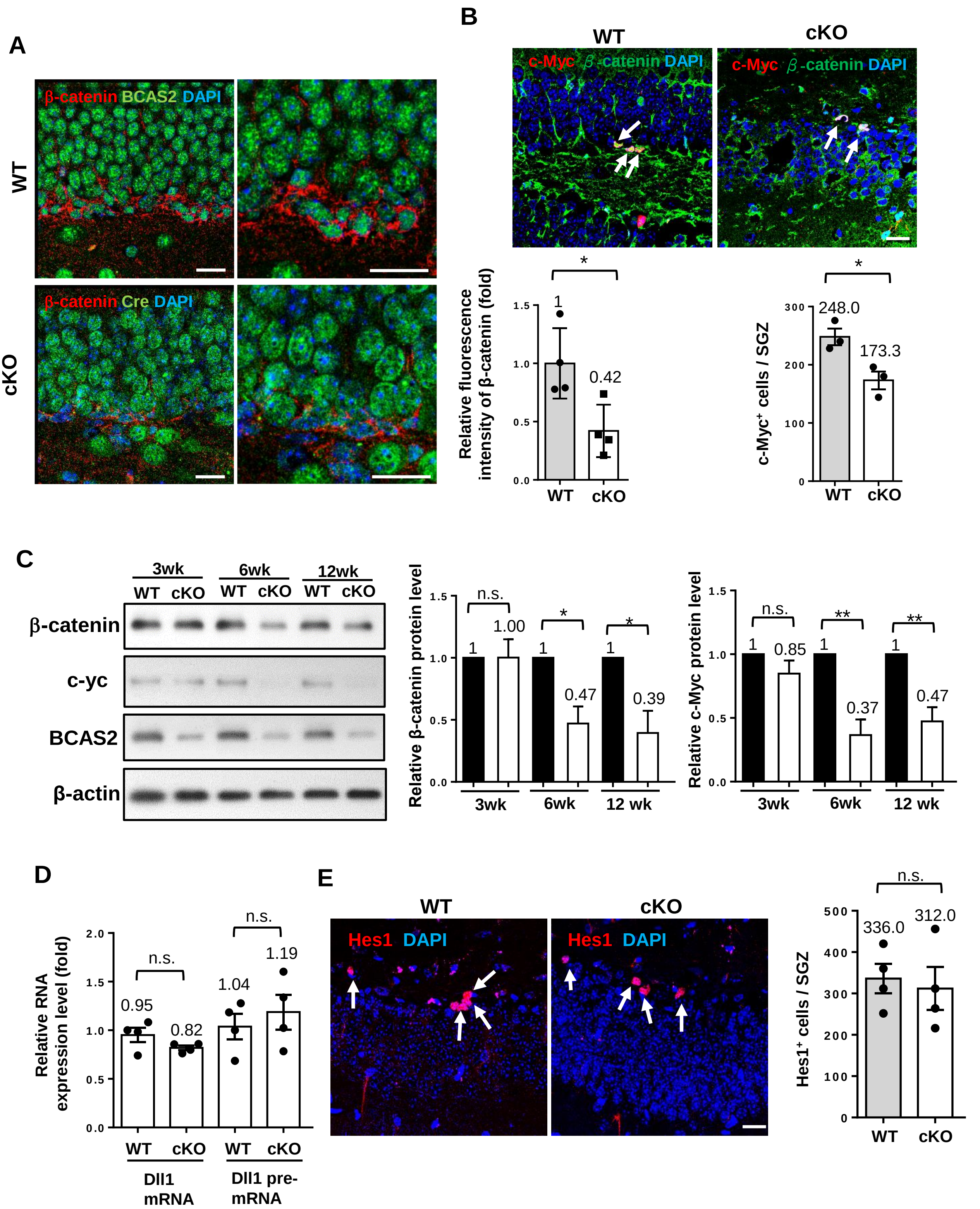

Supplemental Figure 6

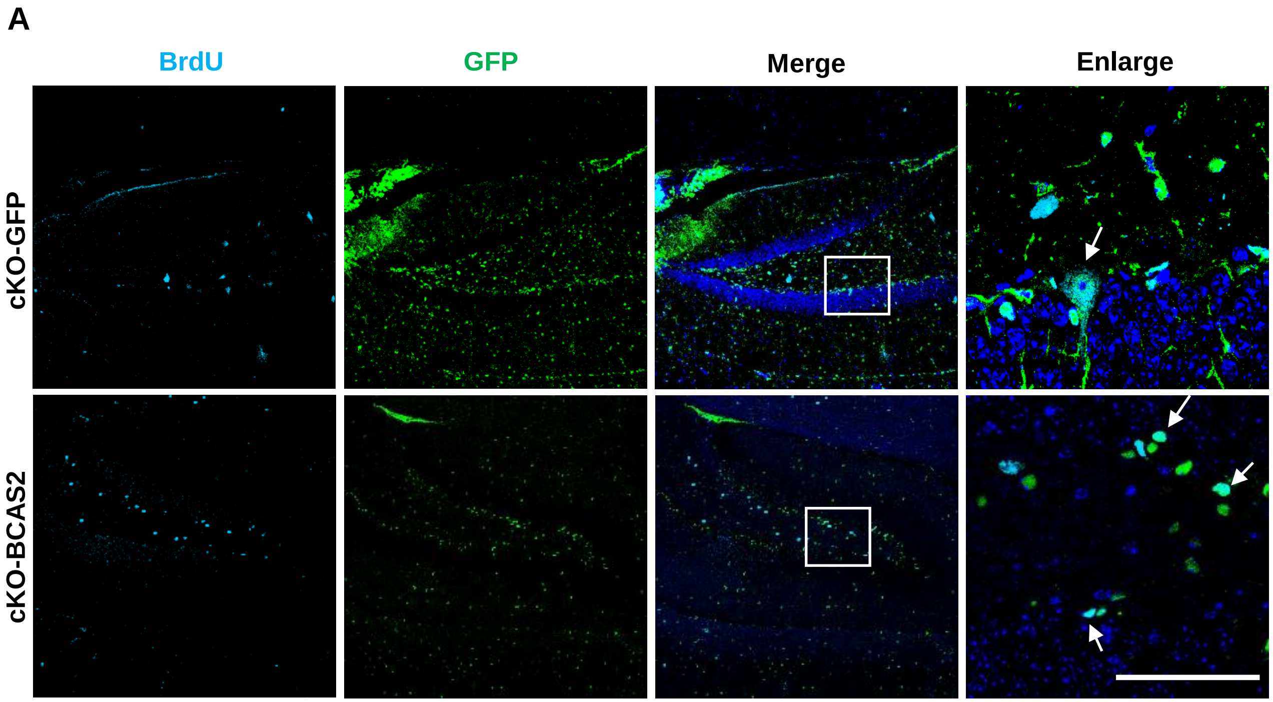

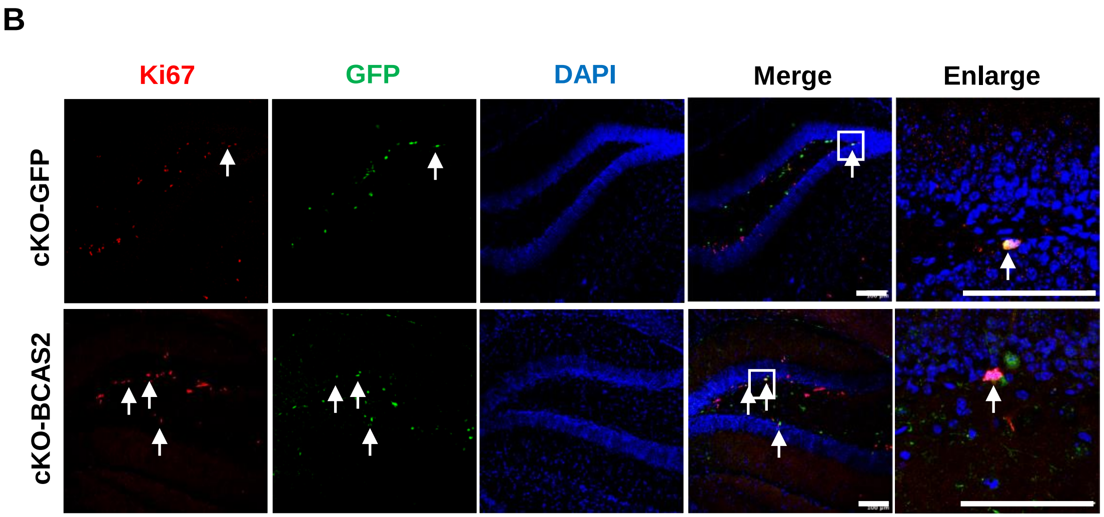

Supplemental Figure 7

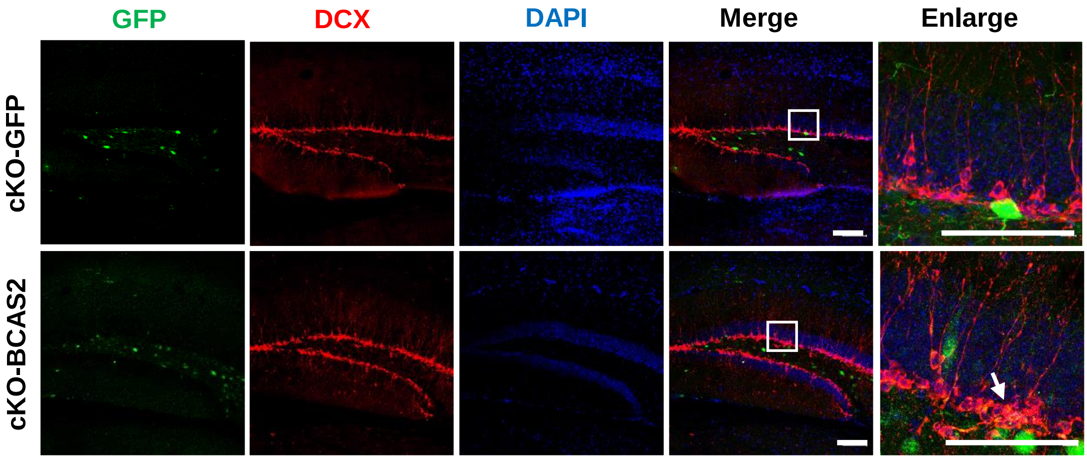

Supplemental Figure 8

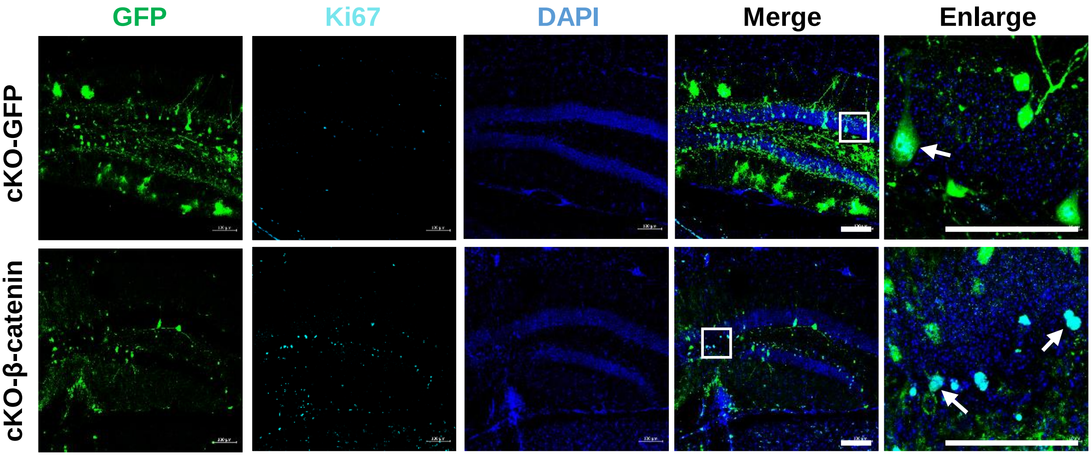

Supplement: Supplementary file 1 — Additional file 1. Supplemental Figures. [file 13287_2022_2837_MOESM1_ESM.pdf]
