# Supplementary material for: Breast carcinoma-amplified sequence 2 regulates adult neurogenesis via β-catenin
Source: Stem Cell Res Ther. 2022 Apr 11;13:160. doi: 10.1186/s13287-022-02837-9 (PMC8996563; doi:10.1186/s13287-022-02837-9)
Supplement: Supplementary file 2 — Additional file 2. Supplemental Table. [file 13287_2022_2837_MOESM2_ESM.docx]

**Table S1.** Antibodies used in the study.

| **Antibody** | **Company & Cat. No.** | **Antibody** | **Company & Cat. No.** |
| --- | --- | --- | --- |
| Cre recombinase | Abcam; ab190177 | BCAS2 | Proteintech; 10414-1-AP |
| NeuN | Millipore; MABN140 | β-catenin | BD; 610154 |
| Sox2 | Abcam; ab79351 | Mouse IgG | Sigma Aldrich; A9044 |
| BrdU | Abcam; ab6326 | Rabbit IgG | Sigma Aldrich; A0545 |
| β-actin | Sigma-Aldrich; A5441 | GSK-3β | BD; 610154 |
| DCX | Millipore; AB2253 | GFAP | Sigma Aldrich; G3893 |
| GAD67 | Abcam; ab183999 | Caspase-3 | Cleaved Cell signaling; 9661 |
| c-Myc | Abcam; ab152146 |  |  |
